# Juvenile idiopathic arthritis managed in the new millennium: one year outcomes of an inception cohort of Australian children

**DOI:** 10.1186/s12969-018-0288-z

**Published:** 2018-11-09

**Authors:** Georgina Tiller, Joanne Buckle, Roger Allen, Jane Munro, Peter Gowdie, Angela Cox, Jonathan Akikusa

**Affiliations:** 1 Department of Rheumatology, The Royal Children’s Hospital, 50 Flemington Rd, Parkville, Melbourne, VIC 3052 Australia; 2 Murdoch Childrens Research Institute, Melbourne, Australia

**Keywords:** Juvenile idiopathic arthritis, Cohort, Outcomes

## Abstract

**Background:**

The advent of new treatments for Juvenile Idiopathic Arthritis (JIA) has prompted interest in systematically studying the outcomes of patients treated in the ‘modern era’. Such data provide both benchmarks for assessing local outcomes and important information for use in counselling families of newly diagnosed patients. While data are available for cohorts in Europe and North America, no such data exist for Australian patients.

The aim was to examine the demographics, treatment and outcomes at 12 months of an inception cohort of newly diagnosed patients with JIA at a single tertiary referral paediatric rheumatology centre in Australia.

**Methods:**

Retrospective review of prospectively collected data from patients newly diagnosed with JIA between 2010 and 2014 at the Royal Children’s Hospital in Melbourne.

**Results:**

One hundred thirty four patients were included (62% female). Oligoarthritis was the single largest category of JIA (36%) and rheumatoid factor positive polyarthritis the least common (2%). Undifferentiated JIA accounted for 13% of patients and was the third largest category. Across the cohort 94% received NSAIDs, 53% oral steroids, 62% methotrexate and 15% a biologic DMARD. Intra-articular steroids were used in 62%, most commonly in the oligoarticular subtype (94%). 95% of patients achieved a joint count of zero at a median of 4.1 months, however flares occurred in 42%. At 12 months 65% had no active joint disease, though more than half remained on medication.

**Conclusion:**

Australian children with JIA managed in the modern era have similar characteristics and achieve short term outcomes comparable to cohorts in Europe and North America, with high rates of joint remission in the first 12 months of follow-up but with a significant relapse rate and requirement for ongoing medication.

## Introduction

Juvenile idiopathic arthritis (JIA) is a heterogeneous group of conditions, characterized by the onset of persistent arthritis before the 16th birthday for which no cause can be identified [1]. It is the most common inflammatory rheumatic disease in childhood [2]. Recent reports of large prospective cohorts of patients with JIA from North America and Europe have led to an improved understanding of the early disease course, contemporary management and short-term outcomes of this condition [3–6]. In addition, these and other studies have contributed to the understanding that early disease course and response to treatment may predict outcome more accurately than disease category, making studies focused on the early disease period in the era of contemporary treatments increasingly important [7, 8].

In Australia, the estimated overall prevalence of JIA is 1–4 per 1000 children although national statistics and publications are limited [9, 10]. Despite its importance and relative frequency, there are no studies describing the outcome of children with JIA in Australia managed with current treatments. The aim of this study was to describe the clinical features, management, course and outcomes during the first 12 months of follow up, for an inception cohort of Australian children with JIA.

## Patients and methods

We conducted a retrospective review of data from an inception cohort of children with JIA diagnosed between October 2010 and October 2014 at The Royal Children’s Hospital (RCH), Melbourne, Australia. All children newly diagnosed with JIA during the relevant period and whose subsequent follow-up had been at the RCH were eligible for inclusion. All data had been prospectively documented in the Rheumatology Department database, a comprehensive clinical tool coded in Microsoft Access™ in which patient demographic details, diagnoses, clinic visits, medications, elective admissions for procedures and communications are recorded. At our institution, patients with a new diagnosis of JIA are followed routinely at least every three months for the first 12–18 months of their arthritis course. For the purposes of this study, patients followed less frequently than this over the relevant period were considered to have incomplete follow-up and were excluded. Acute hospital admissions between scheduled appointments were not included in this analysis.

For ease of analysis, data from the Rheumatology database were exported into an Excel ™ workbook. Exported data fields included patient demographics, assigned JIA category, medications, procedures, clinic visit dates and active joint count at each visit. The JIA category assigned by the treating rheumatologist was confirmed by the primary investigator (GT) using the International League of Associations for Rheumatology (ILAR) classification criteria. For the systemic category, where the ILAR classification was not met due to absence of active arthritis, the diagnosis was made by the treating paediatric rheumatologist. For the purposes of analysis, the two oligoarthritis categories (i.e. persistent and extended) were combined due to a small number of patients in the extended category.

The first visit was defined as the date of diagnosis of JIA. The 12-month visit was the date closest to 12 months from the date of first visit within a 2-month window. The time to zero joint count was calculated as the interval between the first visit and the date on which the treating rheumatologist recorded an active joint count of 0, which for the purposes of this study was considered to represent inactive arthritis. The date of first arthritis flare was the visit date at which a joint count was recorded as > 0 following the first visit at which the joint count had been 0. Uveitis at diagnosis was defined as uveitis detected prior to, or within one month of the diagnosis of JIA. The date of a first episode of uveitis was the visit at which uveitis was first documented by an ophthalmologist. Uveitis was recorded if present at any time during the 12 months of follow up and at the 12-month visit. For patients who did not reach examined outcomes in the first year of follow-up, additional data were collected up to the time at which the relevant outcome occurred or 36 months, whichever came first.

Medications were grouped as non-steroidal anti-inflammatories (NSAIDS), conventional disease modifying agents (cDMARDS) (ie. methotrexate, sulfasalazine, leflunomide), biologic disease modifying agents (bDMARDS) (ie. adalimumab, etanercept, infliximab, tocilizumab, anakinra), oral, intravenous (IV) and ophthalmic steroids. Medications were recorded as ever used in the 12 month follow up period, and in use at the 12 month visit. For cDMARDS and bDMARDS the time from first visit to commencement was calculated in months. Intra-articular steroid (IAS) injections were recorded as the number of visits for joint injections in the 12-month period of follow up. The time to first joint injection was calculated in months. Analysis was performed using descriptive statistics. Survival curves were used to examine medication exposure across the cohort.

The Royal Children’s Hospital (RCH) Human Research Ethics Committee approved the study.

## Results

One hundred sixty one patients were identified, of whom, 27 were excluded because of incomplete follow-up. Demographic and disease details of the 161 patients are given in Table 1. Of the 17 undifferentiated patients: 7 were males > 6 years, HLAB27 positive with a family history of psoriasis; 8 were oligo or poly arthritis with a family history of psoriasis with no other features; 2 were oligoarthritis with B27 and rheumatoid factor (RF) positivity. There were no statistically significant differences in age at diagnosis, gender and proportion of patients when comparing included and excluded patients.

**Table 1:** Demographics of the excluded patients and cohort presented in decreasing order of frequency of JIA category

	Number of excluded patients n (%)	Mean age excluded patients at diagnosis (years)	Female excluded patients n (%)	Number of included patients n (%)	Median age included patients at diagnosis (years)	Female included patients n (%)	Pre-diagnosis symptom period average months (range)	Median joint count baseline (range)	ANA positive n/n done (%)
Oligoarticular	12 (44)	7.5	7 (58)	48 (36)	3.4	34 (71)	3.9 (0.3–24.0)	2 (1–4)	36/47 (77)
Polyarticular RF-	1(4)	9.9	0 (0)	34 (25)	9.0	26 (76)	5.3 (0.5–24.0)	8 (1–42)	21/33 (64)
Undifferentiated	8 (30)	8.4	4 (50)	17 (13)	12.3	7 (41)	7.4 (0.5–24.0)	5 (1–43)	8/16 (50)
Enthesitis related arthritis	2(7)	10.8	0 (0)	13 (10)	13.9	1 (8)	11.2 (0.5–48.0)	2 (1–18)	0/7 (0)
Systemic	1(4)	3.2	0 (0)	10 (7)	8.4	7 (70)	1.4 (0.3–4.0)	5 (0–22)	3/8 (38)
Psoriatic arthritis	3(11)	11	1 (33)	9 (7)	9.3	5 (56)	9.9 (1.5–24.0)	5 (1–36)	2/7 (29)
Polyarticular RF +	0(0)	n/a	n/a	3 (2)	15.6	3 (100)	7.5 (3.8–12.1)	10 (7–44)	2/3 (67)
Overall	27	9.2	12 (44)	134	8.3	83 (62)	5.7 (0.3–48.0)	2 (0–44)	72/121 (60)

### Disease course

Details of the disease course of the cohort over the study period are provided in Table 2. In the 12 months following diagnosis, 127 (95%) of patients achieved an active joint count of zero on at least one occasion. The median time to achieve this outcome within the first 12 months was 3.1 months (Range 0.4–13.1 months) (Table 2). For the 7 (5%) who did not reach a joint count of zero, the median time to a joint count of zero was 18.5 months (range 15.2–32.0 months). Of this group, 4 (57%) were in the polyarticular RF negative JIA category. One patient, in the undifferentiated category, a 14 year old female with ANA and RF positive oligoarthritis, treated with two joint injections and methotrexate in the first 12 months, did not attain a zero-joint count during the 36 months of available follow up data.

**Table 2:** Metrics of arthritis and uveitis activity of the cohort by JIA category for outcomes attained in the first twelve months

	Reached zero joint count in first year n (% of subtype)	Median time to zero joint count in the first twelve months (range)	Median joint count at 12 months (range)	Flared ever during first 12 months n (%)	Mdn time to flare from zero joint count (months)	Uveitis during first 12 months n (%)	Active uveitis at 12 months n (%)
Oligoarticular	48 (100)	2.8 (1.0–12.6)	0 (0–4)	15 (31)	3.2	8 (17)	5 (10)
Polyarticular RF -	30 (88)	5.3 (1.1–12.8)	0 (0–14)	16 (47)	3.5	0 (0)	0 (0)
Undifferentiated	16 (94)	3.9 (0.6–13.1)	0 (0–7)	11 (65)	3.9	0 (0)	0 (0)
Enthesitis related arthritis	11 (85)	6.6 (1.4–12.0)	1 (0–15)	5 (38)	5.5	0 (0)	0 (0)
Systemic	10 (100)	1.7 (0.4–8.1)	0 (0–8)	5 (50)	1.2	0 (0)	0 (0)
Psoriatic arthritis	9 (100)	7.6 (1.2–12.2)	0 (0–24)	4 (44)	3.1	1 (11)	1 (11)
Polyarticular RF +	3 (100)	5.9 (3.7–7.0)	0 (0–0)	0 (0)	n/a	0 (0)	0 (0)
Overall	127 (95)	3.1 (0.4–13.1)	0 (0–24)	56 (42)	3.6	9 (7)	6 (4)

Fifty six subjects (42%) had at least one arthritis flare from a joint count of zero. The median number of flares was 1 and the median time to flare was 3.6 months (range 1.2–5.5 months). Of those patients that flared within the first 12 months, 30 (54%) were on a disease modifying medication at the time of the flare, most commonly methotrexate, 26 (50%).The JIA category with the highest proportion of arthritis flares was the undifferentiated group with 65% of patients flaring over the first 12 months. Where patients had first arthritis flares after the first 12 months of follow up, the median time to flare from zero joint count was 13.5 months and from first visit was 20.5 months. All the RF positive polyarthritis patients had arthritis flares, although none within the first 12 months of follow up. In this group, the median time to arthritis flare from a joint count of zero was 17.0 months (range 11.0–25.5 months). Of the 45 (34%) patients who never had an arthritis flare, 53% were in the oligoarthritis category. The proportion of oligoarthritis patients who had no arthritis flares in the first 12 months was 69%.

Uveitis occurred in 2 (1%) patients at diagnosis, 9 (7%) patients in the first 12 months of follow up. 89% of these patients were in the oligoarticular category. 100% of the patients with uveitis were ANA positive.

### Medications

**Fig. 1:**
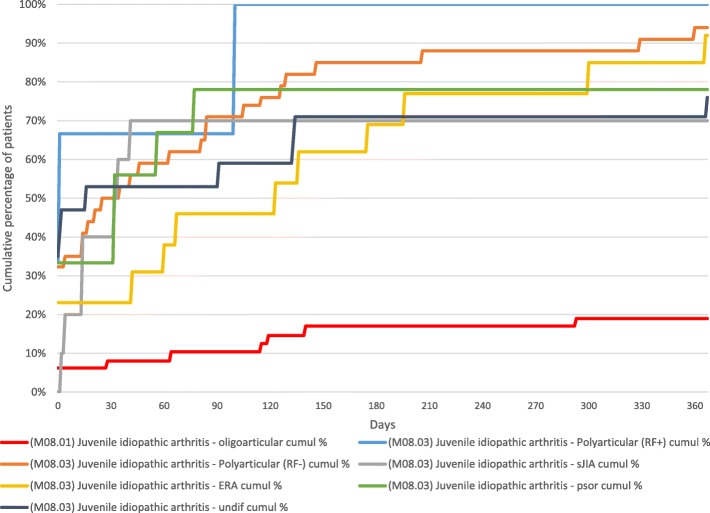
Time to methotrexate commencement by JIA category

Table 3 details the exposure of the cohort to medications over the study period. Non-steroidal anti-inflammatory drugs were almost universally used (94%). Seventy-one patients (53%) were treated with oral corticosteroids at least once and 10 (7%) were treated with intravenous steroids. The median initial dose of oral steroids was 1 mg/kg/day (range 0.2–2) and the median duration of oral steroids, including the completed taper was 4 months (range 0.25–12 months). Eighty-three (62%) were treated with methotrexate, by far the most common cDMARD. Two patients were treated with sulfasalazine in the first 12 months, both of whom had also been treated with methotrexate. Across the whole cohort the median time to commencement of methotrexate was 0.9 months (Table 3). It was shortest in the RF positive polyarthritis category (median 0 months) and longest in patients with enthesitis related arthritis (ERA) (median 3.1 months) (Table 3, Fig. 1). Twenty patients (15%) were commenced on a bDMARD at a median of 5.5 months (Table 3). The patients with the highest rate of bDMARD commencement were in the polyarticular RF positive (33%), ERA (31%) and systemic (sJIA) (30%) categories. All patients with uveitis were treated with ophthalmic steroids during the first 12 months of follow up. Five (56%) were additionally commenced on methotrexate, either for arthritis or uveitis or both. None required addition of a bDMARD within that timeframe.

**Table 3:** Medication exposure during first 12 months and at 12 months by JIA category

	Any	NSAIDS		Oral steroids		IV steroids	Methotrexate			bDMARD			Ocular steroids		Steroid joint injections	
	Ever n (%)	Ever n (%)	At 12 m n (%)	Ever n (%)	At 12 m n (%)	Number given IV steroids n(%)	Ever n (%)	Med. time to (m)	At 12 m n (%)	Ever n (%)	Med. time to (m)	At 12 m n (%)	Ever n (%)	At 12 m n (%)	Ever n (%)	Med joint injections per patient^a^ (range)
Oligoarticular	43(90)	43 (90)	13 (27)	7 (15)	4 (8)	0 (0)	9 (19)	2.1	9 (19)	1 (2)	12.2	1 (2)	8(17)	4(8)	45 (94)	1 (0–3)
Polyarticular RF -	34 (100)	33 (97)	17 (50)	21 (62)	5 (15)	2 (6)	32 (94)	0.8	32 (94)	6 (18)	5.4	6 (18)	0 (0)	0 (0)	16 (47)	0 (0–4)
Undifferentiated	17 (100)	17 (100)	12 (71)	13 (76)	5 (29)	1 (6)	13 (76)	0	12 (71)	3 (18)	5.5	3 (18)	0 (0)	0 (0)	10 (59)	1 (0–2)
Enthesitis related arthritis	13 (100)	13 (100)	7 (54)	10 (76)	4 (31)	0 (0)	12 (92)	3.1	8 (62)	4 (31)	5.8	4 (31)	0 (0)	0 (0)	5 (38)	0 (0–3)
Systemic	10 (100)	9 (90)	5 (50)	10 (100)	3 (30)	6 (60)	7 (70)	0.5	6 (60)	3 (30)	2.4	2 (20)	0 (0)	0 (0)	2 (20)	0 (0–1)
Psoriatic arthritis	9 (100)	8 (89)	5 (56)	7 (78)	2 (22)	0 (0)	7 (78)	1.1	7 (78)	2 (22)	6.0	2 (22)	1 (11)	1 (11)	5 (56)	1 (0–1)
Polyarticular RF +	3 (100)	3 (100)	1 (33)	3 (100)	0 (0)	1 (33)	3 (100)	0	3 (100)	1 (33)	4.7	1 (33)	0 (0)	0 (0)	0 (0)	0 (0–0)
Overall	129 (96)	126 (94)	60 (45)	71 (53)	23 (17)	10 (7)	83 (62)	0.9	77 (57)	20 (15)	5.5	19 (14)	9 (7)	5 (4)	83 (62)	0 (0–4)

Key: *Med*. median; *NSAIDS* non-steroidal anti-inflammatory drugs; *bDMARD* biologic disease modifying anti-rheumatic drug

^a^Median number of elective admissions for joint injections per patient

**Fig. 2:**
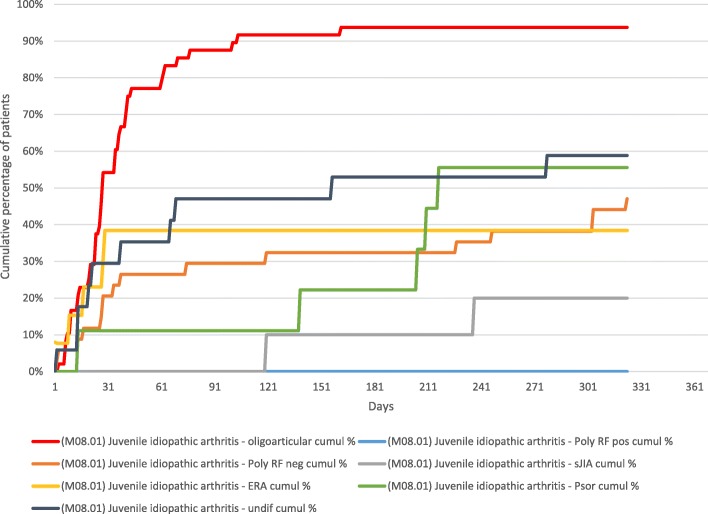
Time to first intra-articular corticosteroid injection by JIA category

Sixty two percent of patients underwent at least one joint injection in the first year of follow up; the median time to first joint injection was 0.9 months (Range 0–11 months). Fifty four percent of patients undergoing intra-articular steroid therapy were in the oligoarticular category, 94% of whom had at least one joint injection in the year following diagnosis (Fig. 2). Ten patients (7%) required their first joint injection after the first 12 months of follow up. The median time to joint injection for this group was 21 months. Seven of these 10 patients were in the RF negative polyarthritis JIA category.

### Outcome at twelve months

Seventy three percent of the cohort were on at least one medication at 12 months, with the two most common being methotrexate (79%) and NSAIDS (62%). Twenty-three (17%) were on oral corticosteroids. The JIA categories with the highest oral steroid use at 12 months were ERA (31%), sJIA(30%), and undifferentiated (29%). All but one patient (with systemic disease) commenced on a bDMARD remained on a bDMARD at 12 months.

**Fig. 3:**
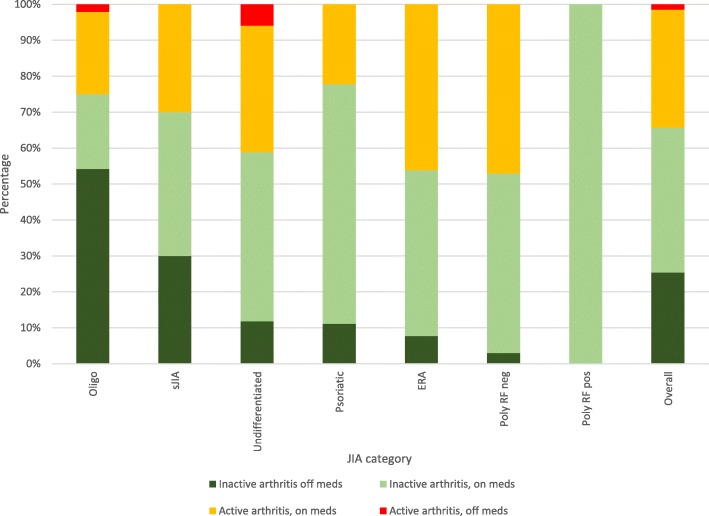
Arthritis activity status by medication use at 1 year according to JIA category

Figure 3 outlines arthritis activity status according to medication use for each JIA category at the 12 month visit. Eighty-eight (66%) of patients had inactive arthritis. Thirty-six (41%) of these patients were of the oligoarticular subtype, of whom 75% had inactive arthritis at this time point. Of the 88 patients with inactive arthritis, 61% were on medications and 39% were off medication. All the RF factor positive polyarticular patients had inactive arthritis on medication at 12 months. Four patients (7%) with inactive arthritis at 12 months had active uveitis.

Of the 46 patients with active arthritis at 12 months, 96% were on medication. The two patients not on medication were awaiting intra-articular therapy. The categories with the highest proportion of patients with active arthritis at 12 months were, polyarticular RF negative (47%) and ERA (46%).

Six patients (4%) had active uveitis at 12 months: 2 had concurrent active arthritis on medication and 4 had inactive arthritis on medications.

## Discussion

This study is the first report of early outcomes for Australian children newly diagnosed with JIA managed in the modern era of arthritis therapy. It demonstrates that Australian children with JIA have similar demographic features, disease course and medication exposure to those reported in large cohorts from Europe and North America [2–5] .

The sex distribution, proportion of patients within each JIA category and prevalence of ANA positivity concur with previously published cohorts [2, 4, 5]. Consistent with previous cohorts, the shortest and longest times from symptom onset to diagnosis were in the sJIA and ERA categories respectively [3, 11]. This likely reflects the common presentation of sJIA as an acute febrile illness, in contrast to the less dramatic symptoms of ERA, typically in adolescent boys, which may be incorrectly interpreted as non-inflammatory musculoskeletal pain or even ignored by the patients themselves. As expected, the systemic category was the only JIA category in which some patients had a zero joint count at diagnosis. It is well recognized that systemic features may precede joint disease in sJIA [12].

Most patients (95%) in our cohort achieved a joint count of zero in the first 12 months of follow up. This outcome was achieved relatively early, at a median time from diagnosis of 4.1 months. These figures are broadly comparable to those in the ReACCh-Out cohort in which ~ 91% of children achieved a zero joint count at a median of 7 months [5]. The slightly greater median time to zero joint count in that cohort may in part relate to the longer period over which this outcome was potentially captured [5]. The time to zero joint count was shortest in the sJIA and oligoarthritis categories (1.7 and 2.8 months, respectively). Early attainment of an active joint count of zero in ssJIA relative to the other categories was also found in the ReACCh-Out cohort [5]. In our patients, this likely reflects the early use of corticosteroids to control systemic disease in patients whose joint disease would not otherwise warrant their use (median joint count at baseline 0, range 0–8). The early response in the oligoarthritis group likely relates to the early use of intra-articular therapy with ~ 90% being injected within three months of diagnosis. This proportion is higher than reported in other cohorts in which rates of intra-articular corticosteroid use were just ~ 50–78% in the first year of follow-up [4, 5]. The higher rate of use of intra-articular steroid use in our patients may relate to ease of access - with 3 scheduled sedation lists and 1 general anaesthetic list per month at our centre - and physician preference. Our results would argue in favour of the early use of this therapy for this group of patients.

A surprising finding in our cohort is that approximately half of all patients received oral corticosteroids in the first year of follow up. This exposure is greater than reported in other cohorts, where oral corticosteroid use in the first year has ranged between 20 and 33% [4, 5, 13]. In our study oral steroids use was captured when an initial dosage was commenced and the date of the completed taper was then recorded. This gives an indication of exposure but makes accurate inferences regarding the cumulative exposure difficult. As expected, steroids were used most commonly in the sJIA and ERA categories and intravenous steroid use was most common in systemic disease. Oral steroids were used in significantly symptomatic patients as a ‘bridging’ measure while awaiting onset of action of DMARDS, or for joint injections. There were clear variations in practice relating to their use between clinicians, with rates of exposure per clinician patient group ranging between 25 and 50% (data not shown). Interestingly, despite the relatively high ‘ever used’ oral steroid exposure found in our cohort, the proportion of patients on oral steroids at 12 months (17%) was similar to the 14% reported in the German JIA cohort [4] and would be consistent with their short-term rather than long-term use.

The use of cDMARDS in our cohort was common; 63% of patients were commenced on a cDMARD in their first year of follow-up including 84% percent of non-oligoarticular and 25% of oligoarticular patients. The overall rate of cDMARDs use was broadly similar to those reported in the German (64.7% on methotrexate) and Canadian (47.6% on first DMARD) cohorts at the same time point [4, 5]. Our reported cDMARD use was higher than reported in the Childhood Arthritis and Rheumatology Research Alliance registry (CARRA), although the 30% cDMARD use in this study was in patients with less than 6 months of disease duration [13]. As in other cohorts, methotrexate was the cDMARD most commonly used and was started at a median of 0.7 months in non-oligoarticular disease and 2.1 months in oligoarticular disease, the latter consistent with its introduction following failure to achieve remission post early intra-articular steroid therapy. Fifteen percent of patients were commenced on biologic therapy; this is substantially more than the ~ 5% at 12 months reported in the ReACCH-Out cohort, and lower than the 38% of any biologic use in the newly diagnosed group in the CARRA cohort, but in line with the experience in the German cohort (22%) [4, 5, 13]. The median time to commencement of a bDMARD was 5.5 months, consistent with adherence to the requirements for accessing government-funded bDMARDS in Australia, which requires demonstration of failure to respond to a minimum of 3 months’ methotrexate therapy [14]. 30% of the systemic arthritis group were commenced on a biologic agent, fewer than would be expected in the era of consensus treatment plans for systemic arthritis [13, 15]. This is likely multifactorial, reflecting a changing practice pattern as new guidelines and data emerge on the first line use of biologics in sJIA, and restrictions of access to biologic treatments for sJIA in Australia particularly at the start of data collection for this study [16]. Taken together these data are consistent with early aggressive treatment of JIA in the modern era, an approach based on predominantly adult data suggesting that early disease control improves long term outcomes in arthritis [17–19]. While the majority of our cohort achieved a zero joint count in the first year of follow-up, a significant minority (42%) subsequently flared. Despite this, across the cohort 65% of patients had inactive joint disease at 12 months although more than half remained on medication. Inactive joint disease off medication was uncommon (25%) except in patients with oligoarthritis (50%) and sJIA (30%), which were the two JIA categories most likely to experience a period of inactive disease in the German cohort [4]. These data suggest modern therapies in JIA are effective in controlling active joint disease in the majority of patients but that the underlying tendency to relapse - and therefore need for ongoing treatment - remains high.

The findings of this study should be interpreted taking into consideration several limitations. Compared to the cohorts in Germany and Canada our sample size is relatively small and from a single centre. Patient related outcomes, such as pain and quality of life, were not included as these data were not available. Similarly, disease remission as per the widely used ‘Wallace criteria’ [20] was not used as an outcome, because components of these criteria (such as acute phase reactants and physician global assessment of disease activity) are not routinely collected in every patient at every visit at our centre. Instead we chose to focus on active joint count which is documented at every visit and which, pragmatically, at most centres is the primary variable that drives contemporaneous treatment decisions in the majority of patients [21, 22]. Using the Juvenile Arthritis Disease Activity Score (JADAS) as an outcome measure, as has been done in other cohorts, may have strengthened our findings, and, we suspect, would have led to lower reported rates of inactive disease in our cohort [6, 22].

Our study also has a number of strengths. It provides ‘real world’ outcome data from consecutively diagnosed patients with JIA managed in a general rheumatology clinic at the largest paediatric rheumatology centre in Australia. Data were prospectively collected in a dedicated database used to record patient encounters in the clinic and, for each patient, joint counts were conducted by the same clinicians over time, negating the risk of inter-observer variability. The paediatric rheumatology department includes five rheumatologists, majority trained in North America, with experience ranging from 5 to 30 years, along with one supervised fellow. Finally, the primary outcome examined - zero joint count- is a concept that is easy to convey when discussing likely outcomes at the start of treatment, making our outcome data directly relevant to patients and their families.

## Conclusions

We have shown that Australian children with JIA managed in the modern era have similar characteristics and achieve short term outcomes comparable to cohorts in North America and Europe. Whether their longer-term outcomes are similar is unknown. While the majority of patients achieve a zero joint count at least once within the first year of follow-up, relapses are common such that one third have active arthritis and three quarters require ongoing medication at 12 months. Ongoing research is required to further understand determinants of disease course and optimum management strategies.

## Abbreviations

ANA: Anti-nuclear antibody
DMARD: Disease-modifying anti-rheumatic drug
ERA: Enthesitis related arthritis
IAS: Intra-articular steroid
ILAR: International League of Associations for Rheumatology
JIA: Juvenile idiopathic arthritis
NSAID: Non-steroidal anti-inflammatory drug
RCH: The Royal Children’s Hospital
RF: Rheumatoid factor
sJIA: Systemic juvenile idiopathic arthritis
